# Complex Hydride‐Based Gel Polymer Electrolytes for Rechargeable Ca‐Metal Batteries

**DOI:** 10.1002/advs.202308318

**Published:** 2024-07-03

**Authors:** Takara Shinohara, Kazuaki Kisu, Arunkumar Dorai, Kenji Zushida, Hiroshi Yabu, Shigeyuki Takagi, Shin‐ichi Orimo

**Affiliations:** ^1^ Institute for Materials Research (IMR) Tohoku University Katahira 2‐1‐1 Aoba‐ku Sendai 980‐8577 Japan; ^2^ Ichikawa Research Centre Sumitomo Metal Mining Co. Ltd. Nakakokubun 3‐18‐5 Ichikawa Chiba 272‐8588 Japan; ^3^ College of Engineering Shibaura Institute of Technology 3‐7‐5 Toyosu, Koto‐ku Tokyo 135‐8548 Japan; ^4^ Institute of Multidisciplinary Research for Advanced Materials Tohoku University Katahira 2‐1‐1 Aoba‐ku Sendai 980‐8577 Japan; ^5^ Advanced Institute for Materials Research (AIMR) Tohoku University Katahira 2‐1‐1 Aoba‐ku Sendai 980‐8577 Japan

**Keywords:** calcium metal anodes, dual‐cation batteries, gel‐polymer electrolytes, hydride‐based electrolytes, rechargeable calcium batteries

## Abstract

Rechargeable Ca batteries offer the advantages of high energy density, low cost, and earth‐abundant constituents, presenting a viable alternative to lithium‐ion batteries. However, using polymer electrolytes in practical Ca batteries is not often reported, despite its potential to prevent leakage and preserve battery flexibility. Herein, a Ca(BH_4_)_2_‐based gel‐polymer electrolyte (GPE) is prepared from Ca(BH_4_)_2_ and poly(tetrahydrofuran) (pTHF) and tested its performance in Ca batteries. The electrolyte demonstrates excellent stability against Ca‐metal anodes and high ionic conductivity. The results of infrared spectroscopy and ^1^H and ^11^B NMR indicate that the terminal ─OH groups of pTHF reacted with BH_4_^−^ anions to form B─H─(pTHF)_3_ moieties, achieving cross‐linking and solidification. Cyclic voltammetry measurements indicate the occurrence of reversible Ca plating/stripping. To improve the performance at high current densities, the GPE is supplemented with LiBH_4_ to achieve a lower overpotential in the Ca plating/stripping process. An all‐solid‐state Ca‐metal battery with a dual‐cation (Ca^2+^ and Li^+^) GPE, a Ca‐metal anode, and a Li_4_Ti_5_O_12_ cathode sustained >200 cycles, confirming their feasibility. The results pave the way for further developing lithium salt‐free Ca batteries by developing electrolyte salts with high oxidation stability and optimal electrochemical properties.

## Graphical abstract

Ca‐conducting gel‐polymer electrolytes synthesized via cross‐linking reaction between borohydride anions and pTHF exhibit the reversible long‐term stable Ca plating/stripping property. The addition of LiBH_4_ improves their electrochemical property and enables long‐life (over 200 cycles) battery operation using the gel polymer electrolyte, Ca metal, and Li_4_Ti_5_O_12_ electrodes.

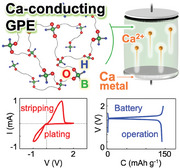

## 1 Introduction

The scarcity of lithium resources and the safety risks posed by conventional lithium‐ion batteries (e.g., the leakage of flammable organic solvent‐based electrolytes) have inspired the development of rechargeable batteries based on the ions of cheap Earth‐abundant multivalent metals such as magnesium, zinc, and calcium.^[^ ^1^ ^]^ In particular, Ca is the fifth most abundant element in the Earth's crust and has a low reduction potential comparable to that of lithium and superior to those of magnesium and zinc (Ca: 4.86 wt.%, −2.87 V vs the standard hydrogen electrode (SHE); Li: 0.0065 wt.%, −3.04 V vs SHE).^[^ ^2^ ^]^ A Ca‐metal anode increases cell voltage due to the metal's lower potential. Moreover, Ca^2+^ ions have a lower charge density than Mg^2+^ and Zn^2+^ ions, engaging in weaker Coulombic interactions with anions and featuring a higher migration rate.^[^ ^3^ ^]^ Because of the above, rechargeable Ca batteries hold significant potential as next‐generation sustainable power sources but have not yet been commercialized because of the instability of most liquid electrolytes against Ca metal.^[^ ^4^ ^]^

Ponrouch et al. (2016)^[^ ^5^ ^]^ reported the first Ca electrolyte (a solution of Ca(BF_4_)_2_ in a mixture of ethylene carbonate (EC) and propylene carbonate (PC)) supporting reversible Ca plating/stripping at high temperatures and showed that repeated plating/stripping decreased Coulombic efficiency by promoting the formation of CaF_2_ and its deposition on the copper substrate. Wang et al. (2018)^[^ ^6^ ^]^ used a solution of Ca(BH_4_)_2_ in tetrahydrofuran (THF) as an electrolyte to achieve reversible Ca plating/stripping at room temperature as well as high first‐deposition Coulombic efficiency (94.8%) and low overpotential (25 mV). This behavior was ascribed to the reaction of Ca(BH_4_)_2_ with THF, affording a small amount of CaH_2_, which formed a protective layer on the Ca‐metal electrode and prevented the generation of Ca plating/stripping inhibitors such as CaCO_3_ and Ca(OH)_2_. Other liquid electrolytes with high oxidation stability have also been developed;^[^ ^7^ ^]^ however, works aiming to optimize Ca plating/stripping have not reported electrolytes beyond those based on Ca(BH_4_)_2_.

Compared with conventional liquid electrolytes, solid‐polymer electrolytes (SPEs) and gel‐polymer electrolytes (GPEs) are less prone to short‐circuiting, dendrite growth, and leakage, thus posing lower safety risks.^[^ ^8^ ^]^ Polymer electrolytes allow the use of lithium anodes, thus improving battery capacity by suppressing dendritic growth. Electrolytes with high mechanical properties (such as shear modulus) are known to be effective in suppressing dendrite growth.^[^ ^9^ ^]^ For example, it has been reported that dendrite growth can be suppressed by using block copolymers containing two types of monomers,^[^ ^10^ ^]^ polymer electrolytes synthesized using cross‐linking reactions,^[^ ^11^ ^]^ and the addition of inorganic fillers.^[^ ^12^ ^]^ In addition, due to the absence of liquid leakage, the risk of ignition from flammable organic electrolytes can be minimized. Vanitha et al. (2018)^[^ ^13^ ^]^ obtained an SPE with high ionic conductivity of 1.736 × 10^−4^ S cm^−1^ by drying a solution of CaCl_2_ in a mixture of poly(vinyl alcohol) and poly(vinyl pyrrolidone). Hosein's group reported SPEs based on [Ca(NO_3_)_2_ + poly(ethylene glycol) diacrylate (PEGDA)]^[^ ^14^ ^]^ and [Ca(NO_3_)_2_ + poly(THF) (pTHF) + epoxy]^[^ ^15^ ^]^ combinations as well as GPEs based on the combinations of [Ca(BF_4_)_2_ + PEGDA/1‐ethyl‐3‐methylimidazolium trifluoromethanesulfonate (solvent)],^[^ ^16^ ^]^ [Ca(BF_4_)_2_ or Ca(TFSI)_2_ or Ca(ClO_4_)_2_ + PEGDA/EC + PC],^[^ ^17^ ^]^ and [Ca(TFSI)_2_ + poly(vinylimidazole)/1‐vinylimidazole].^[^ ^18^ ^]^ These electrolytes showed good conductivities (10^−3^–10^−6^ S cm^−1^), with one supporting charge/discharge in a Ca_3_Co_4_O_9_||V_2_O_5_ cell.^[^ ^16^ ^]^ Despite their availability, highly Ca ion–conductive polymer electrolytes require optimization in terms of stability against Ca metal and durability during prolonged use in rechargeable batteries.

To address this problem, several researchers have focused on complex hydride salts^[^ ^19^ ^]^ and pTHF‐based polymers that exhibit high reduction stability.^[^ ^20^ ^]^ They are therefore compatible with metal (e.g., lithium,^[^ ^21^ ^]^ sodium,^[^ ^22^ ^]^ magnesium,^[^ ^23^ ^]^ zinc,^[^ ^24^ ^]^ and calcium^[^ ^7^, ^25^ ^]^) anodes. Green et al. described a high‐performance GPE composed of LiB_12_H_12_, PC, and poly(methyl methacrylate) in their work on using hydride salts as polymer electrolyte components.^[^ ^26^ ^]^ Du et al. focused on electrolytes with complex hydride salts of divalent metals, revealing that the GPE prepared via an in situ cross‐linking reaction between Mg(BH_4_)_2_ and the terminal ─OH groups of pTHF supported reversible magnesium plating/stripping at room temperature while featuring high magnesium‐ion conductivity and high stability against magnesium metal.^[^ ^27^ ^]^

Inspired by the above, we herein synthesized a GPE with high Ca ion conductivity and long‐term stability against Ca metal via the cross‐linking reaction of pTHF with Ca(BH_4_)_2_, a complex hydride compatible with metal anodes because of its high reducibility. Unlike the [Mg(BH_4_)_2_ + pTHF]^[^ ^27^ ^]^ electrolyte described previously, our GPE achieved superior Ca plating/stripping performance without the use of metal chlorides, and the short‐circuit current was significantly reduced compared to the liquid system. In addition, the electrochemical properties of the GPE at high current densities could be improved by adding LiBH_4_. For a practical utility demonstration, a Ca‐metal battery composed of this dual‐cation GPE, a Li_4_Ti_5_O_12_ cathode, and a Ca‐metal anode was subjected to charge/discharge testing.

## 2 Results and Discussion

### 2.1 Synthesis and Structural Characterization of GPE

**Figure 1:**
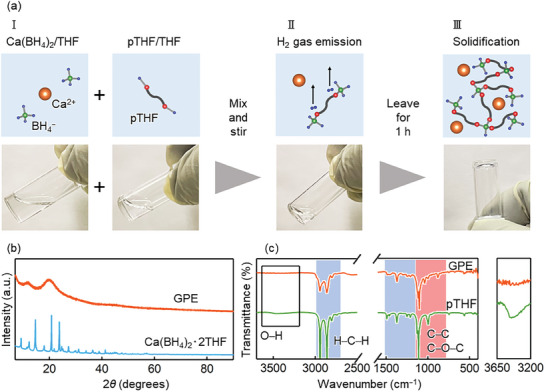
a) Synthesis of calcium‐based gel polymer electrolyte (GPE) and photographs corresponding to different stages of this synthesis. b) XRD patterns of GPE and Ca(BH_4_)_2_·2THF; c) FT‐IR spectra of GPE and pTHF.

During GPE synthesis (**Figure** **1**a), BH_4_^−^ anions reacted with the terminal –OH groups of pTHF to generate H_2_ and a network of borohydride‐linked pTHF chains, which resulted in solidification (i.e., gel polymer formation). This mechanism is nearly identical to that used to form GPE from Mg(BH_4_)_2_ and pTHF.^[^ ^27^ ^]^

To investigate the cross‐linking reaction between BH_4_^−^ anion and terminal ─OH in pTHF and the structure of the synthesized GPE, Fourier transform infrared spectroscopy (FT‐IR), Raman spectroscopy, nuclear magnetic resonance (NMR) spectroscopy, and X‐ray diffraction (XRD) analyses were conducted. The XRD pattern of our GPE (Figure 1b) featured broad peaks indicative of an amorphous structure, and the absence of Ca(BH_4_)_2_ ·2THF peaks showed that this salt was completely dissolved. Figure 1c shows the FT‐IR spectra of pTHF and GPE, with blue and red regions corresponding to the vibrations of H─C─H and C─C/C─O─C moieties in pTHF, respectively.^[^ ^28^ ^]^ The terminal ─OH peak of pTHF ≈3500 cm^−1^ disappeared after treatment with Ca(BH_4_)_2_; that is, the reaction of the terminal ─OH groups and BH_4_^−^ anions proceeded to completion. The related Raman spectra (Figure S1, Supporting Information) revealed that each peak observed for our GPE could be mapped to a peak observed for the GPE prepared from Mg(BH_4_)_2_ and pTHF,^[^ ^27^ ^]^ which suggested that the related reaction mechanisms and structures were similar.

**Figure 2:**
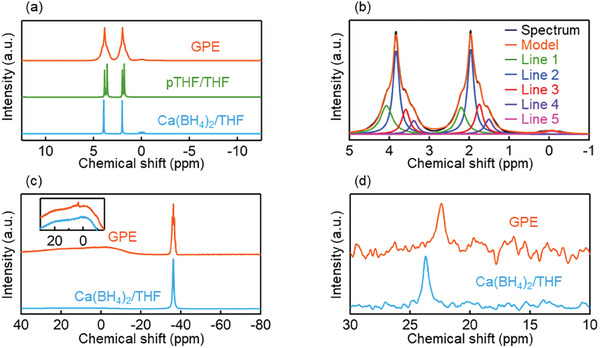
a) ^1^H NMR spectra of solidified GPE, pTHF/THF, and Ca(BH_4_)_2_/THF. b) Deconvoluted ^1^H NMR spectrum of GPE (Line 1: Ca^2+^ coordinated THF, Line 2: free THF, Line 3: free pTHF, Line 4: pTHF coordinated to BH_4_^−^, Line 5: free BH_4_^−^). c) ^11^B NMR spectra of solidified GPE and Ca(BH_4_)_2_/THF. d) ^43^Ca NMR spectra of solidified GPE and Ca(BH_4_)_2_/THF.

**Figure** **2**a presents the ^1^H NMR spectra of the GPE (1 h after cross‐linking), pTHF in THF, and Ca(BH_4_)_2_ in THF. For Ca(BH_4_)_2_ in THF, the peaks at 3.8 and 2 ppm correspond to the protons near and far from the oxygen in THF, respectively, whereas the quartet ≈0 ppm corresponds to BH_4_^−^. For pTHF in THF, the peaks at 3.5 and 1.7 ppm correspond to the protons near and far from the oxygen in pTHF, respectively. In the case of the GPE, the peaks of protons near (≈3.7 ppm) and far (≈1.9 ppm) from oxygen in THF and pTHF were broadened because of the inhibition of molecular motion by polymerization and could be deconvoluted into the contributions of Ca^2+^‐coordinated THF (Line 1), free THF (Line 2), free pTHF (Line 3), and pTHF coordinated to BH_4_^−^ (Line 4) (Figure 2b).

The strong peak at −38 ppm in the ^11^B NMR spectra of Ca(BH_4_)_2_ and GPE was ascribed to BH_4_^−^ (Figure 2c), and the weak peak at 3 ppm observed for GPE was attributed to the B─H─(pTHF)_3_ moieties produced in the initial stage of polymerization via the reaction between BH_4_^−^ and pTHF. This assignment was corroborated by the ^1^H NMR peak of B─H─(pTHF)_3_ at 4.68 ppm observed immediately after polymerization (Figure S2, Supporting Information). Figure S2 (Supporting Information) illustrates that sharp peaks corresponding to THF, pTHF, and BH_4_^−^ were observed in the early stage of polymerization, while separate peaks corresponding to Ca^2+^‐solvating THF and BH_4_^−^–coordinating pTHF were not observed, possibly because of the fast (on the NMR timescale) intermolecular exchange. Moreover, the narrow ^1^H NMR peaks in Figure S2 (Supporting Information) indicate that molecular motion was not inhibited in the early stage of polymerization.

The ^43^Ca NMR peak of the GPE (Figure 2d) was narrow (29 Hz) as one of Ca(BH_4_)_2_ in THF (17 Hz), which suggested that the diffusion and conduction of Ca^2+^ in the GPE were fast. Overall, these results indicated that our GPE synthesis was successful.

### 2.2 Electrochemical Characterization of GPE

**Figure 3:**
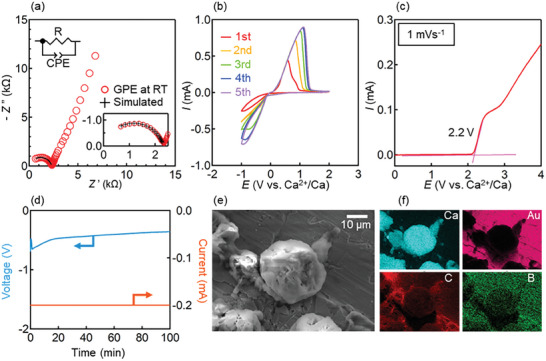
Electrochemical analysis of the GPE. a) Nyquist plot with equivalent circuit and expanded high‐frequency region as insets. b) Cyclic voltammograms were recorded at a scan rate of 10 mV s^−1^. c) Linear sweep voltammogram recorded at a scan rate of 1 mV s^−1^. d) Voltage and current evolution with time during the deposition test. e) Scanning electron microscopy image and f) corresponding elemental mappings (Ca, Au, C, and B) of the deposit formed on the gold electrode.

To verify the occurrence of rapid Ca^2+^ diffusion suggested by NMR analysis, we evaluated the ionic conductivity of our GPE by EIS. The obtained Nyquist plot featured a semicircle in the high‐frequency range derived from the bulk electrolyte and a spike in the low‐frequency range reflecting the electrode contribution due to the ion‐blocking cell (**Figure** **3**a). By fitting the high‐frequency‐range semicircle using the equivalent circuit shown in the inset of Figure 3a, we calculated ion conductivity (*σ*) as 1.92 × 10^−5^ S cm^−1^ at room temperature. The simulated impedance values of the respective frequencies and fitting parameters are shown in the supplementary information (Figure S3 and Table S1, Supporting Information).

The cyclic voltammetry (CV) curve of the Ca|GPE|Au cell featured an anodic peak corresponding to the transition of Ca^2+^ from Ca metal to the gold surface below 0 V and a cathodic peak corresponding to the reverse transition above 0 V, which indicated the occurrence of Ca plating/stripping based on Ca conduction/migration (Figure 3b).^[^ ^29^ ^]^ After the second cycle, the current increase was attributed to conditioning at the electrolyte‐electrode interface, as in the galvanostatic test described below. Similar behavior has been observed for a Mg‐based GPE.^[^ ^30^ ^]^ To the best of our knowledge, no reports regarding calcium plating/stripping have been confirmed using CV in Ca‐based polymer electrolytes so far.^[^ ^13^, ^14^, ^15^, ^16^, ^17^, ^18^ ^]^ Our GPE is the first Ca‐based polymer electrolyte to demonstrate reversible Ca plating/stripping and high stability against a Ca‐metal electrode, according to electrochemical measurements. To investigate the anodic stability of our GPE, we conducted linear sweep voltammetry (LSV) measurements in the configuration used for CV (Figure 3c; Figure S4, Supporting Information). Evaluation of the oxidation stability of electrolytes is important in considering whether high‐voltage cathode active materials can be used in actual charge/discharge reactions. The current exhibited a notable spike at ≈2.2 V, increasing with increasing voltage, which suggested that this voltage corresponded to the onset of the oxidative decomposition of BH_4_^−^ on the gold electrode.^[^ ^6^ ^]^ An oxidative current spike appeared at a much higher voltage on the molybdenum and aluminum electrodes due to the different overvoltages required for the onset of the oxidative decomposition of borohydride, depending on the type of electrode. This may be due to differences in the degree of adsorption of borohydride anions on the electrode surface depending on the type of electrode, resulting in the decomposition of borohydride anions with low oxidation stability and the generation of an oxidation current. Such differences in overvoltage, depending on the electrode type, have also been reported in other electrochemical systems.^[^ ^29^, ^31^ ^]^ CV measurements were performed to investigate if decomposition products decreased plating/stripping cyclability of GPE. The current decreased over cycles (Figure S5, Supporting Information). This indicates that the decomposition product inhibited Ca plating/stripping on the gold electrode.

To confirm the deposition of Ca on the gold electrode, we conducted a constant‐current test (|*I*| = 0.2 mA) using the cell employed for CV testing. The GPE resistance calculated from the voltage and current (Figure 3d) agreed with that extracted from the Nyquist plot. In the disassembled cell, black powders deposited on the gold electrode were observed after washing with THF. The SEM and energy‐dispersive X‐ray spectroscopy analyses of these deposits revealed the presence of uniformly dispersed Ca‐metal particles (Figure 3e,f; Figure S6, Supporting Information). Thus, Ca^2+^ ions were conducted through the GPE, aligning with NMR and CV results.

**Figure 4:**
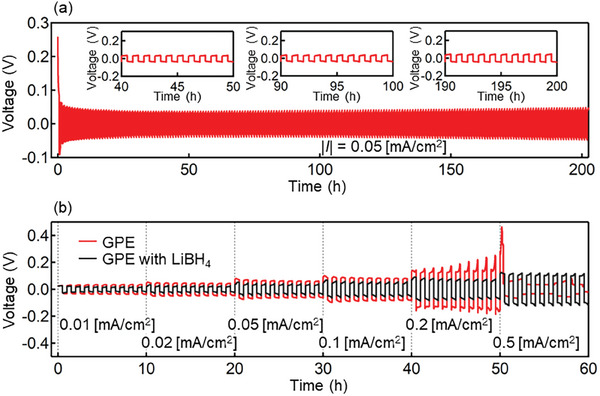
Electrochemical analysis of GPE. a) Galvanostatic curves (|*I*| = 0.05 mA cm^−2^). b) Voltage changes of (red) Ca^2+^‐only and (black) dual‐ion GPEs at various current densities.

Next, Ca plating/stripping cyclability was investigated by galvanostatically cycling a symmetric Ca|GPE|Ca cell at a constant current density of 0.05 mA cm^−2^ (**Figure** **4**a). The symmetric cell could be continuously operated for >200 h, which indicated that the GPE was stable against Ca metal plating/stripping. The initial significant voltage spike originating from the conditioning of the electrolyte/electrode interface,^[^ ^32^ ^]^ during which it is considered that a solid‐electrolyte interface (SEI) forms on the Ca metal electrodes, decreased over several tens of cycles, as has been observed for systems with polymer electrolytes based on other multivalent metals.^[^ ^30^, ^33^ ^]^ Considering that the main component of GPE is Ca(BH_4_)_2_/THF, the SEI in our system is assumed to have the same composition as that reported for the SEI in the Ca(BH_4_)_2_/THF liquid electrolyte system. In the Ca(BH_4_)_2_/THF liquid electrolyte system, the SEI on the Ca metal electrode consists of calcium oxide and calcium carbonate in the borate and organic matrix, where Ca^2+^ ions can migrate.^[^ ^34^ ^]^ Therefore, the SEI in our system likely contains decomposition products of Ca(BH_4_)_2_/THF, which should be the preferred SEI for battery operation. The formation of a SEI in which Ca^2+^ ions can migrate is thought to reduce the initial voltage. This initial voltage spike was observed at a low current density (Figure S7, Supporting Information). The voltage response was a square wave with a symmetry equal to or better than that observed for other Mg‐^[^ ^27^, ^30^, ^33^ ^]^ and Ca‐based^[^ ^17^, ^18^ ^]^ polymer electrolytes (Figure 4a, inset). The voltage response was stable for more than 200 cycles, which indicated that the GPE exhibited stable behavior at the Ca‐metal interface.

The previously reported Mg‐based GPE with a similar design also showed high stability against metal anodes.^[^ ^27^ **^]^** Compared to the Mg‐based GPE, our GPE exhibited high reversibility in the absence of auxiliary salts such as MgCl_2_ and under harsher conditions because of the lower redox potential of Ca metal. This behavior seems to originate from the higher coordinational flexibility of Ca^2+^ compared to that of Mg^2+^ and the formation of ion clusters such as Ca(BH_4_)^+^ and Ca(BH_4_)_3_^−^ in the matrix solvent, which helps to improve Ca plating/stripping on the Ca‐metal electrode.**^[^** ^34^, ^35^ **^]^** Although a previously reported Ca‐based polymer electrolyte featured a higher ionic conductivity than our GPE,^[^ ^18^ **^]^** its overpotential, determined by galvanostatic measurements, was ten times higher. This confirms that our GPE was highly stable against Ca‐metal electrodes, as few by‐products inhibiting Ca plating/stripping were formed in the Ca(BH_4_)_2_/THF electrolyte.**^[^** ^6^, ^34^, ^35^ **^]^** To compare the electrochemical properties depending on the amount of THF content in the electrolyte, the galvanostatic tests under the same conditions as in Figure 4a were performed using THF‐poor and THF‐rich GPE. The overvoltage of initial interface conditioning in THF‐rich GPE was lower than that of the other two GPEs (Figure S8a–c, Supporting Information). Increasing THF content in GPE increased the overvoltage of initial interface conditioning. This indicates that a suitable electrolyte/electrode interface forms in THF‐rich GPE, as in liquid electrolytes. The overvoltage increased gradually with each cycle in THF‐rich GPE, indicating that the excess amount of THF solvent decomposed and its decomposition products inhibited calcium plating/stripping. The overvoltage in the THF‐rich GPE was large, suggesting the salt concentration was low and few electrochemically active species were present. On the other hand, THF‐poor GPE exhibited a large overvoltage of plating/stripping. In the THF‐poor GPE, borohydride anions react with pTHF, whose concentration is high, and the amount of generated H_2_ gas is greater than that of GPE. Such an H_2_ gas bubble on the electrodes is considered to reduce the area where electron transfer and reception reactions can occur, which indicates that the overvoltage is large in THF‐poor GPE.

### 2.3 Dual‐Ion GPE and Battery Application

Figure 4b (red line) illustrates the voltage changes observed when the Ca|GPE|Ca symmetric cell was tested at various current densities. Although the voltage was stable at current densities of up to 0.1 mA cm^−2^, it gradually increased at higher current densities. The low ionic conductivity of our GPE may be caused by forming a solid–electrolyte interphase or side reactions with the solvent. During the first cycle at 0.5 mA cm^−2^, the overvoltage decreased, which was also observed for another electrochemical cell under the same conditions (Figure S9, Supporting Information). A drastic voltage drop was confirmed at random times, indicating the occurrence of micro short circuits. To address this problem, we synthesized a dual‐cation electrolyte containing Ca(BH_4_)_2_ and LiBH_4_. Adding LiBH_4_ (or other monovalent‐cation salts) was reported to improve electrochemical properties and Coulombic efficiency and suppress the increase in overpotential.^[^ ^36^ ^]^ In addition, charge/discharge tests were performed using a Ca‐metal anode and a Li_4_Ti_5_O_12_^[^ ^36b^ ^]^ or FeS_2_^[^ ^36c^ ^]^ cathode in the system featuring LiBH_4_‐supplemented Ca(BH_4_)_2_/THF as the liquid electrolyte.

The dual‐cation GPE with Ca(BH_4_)_2_ and LiBH_4_ was synthesized using the method employed for the Ca‐based GPE and solidified similarly to the latter, as both salts contained the BH_4_^−^ ion that reacted with pTHF. The black line in Figure 4b illustrates the voltage change for the cell with the LiBH_4_‐containing GPE determined under the conditions corresponding to those of the black line. As expected, adding LiBH_4_ suppressed the overpotential increase because LiBH_4_ promoted the Ca^2+^ desolvation process.^[^ ^36b^ ^]^ It has been stated that the coordination number of oxygen around Ca^2+^ in the Ca(BH_4_)_2_+LiBH_4_/THF (dual‐cation) system is lower than that in the pure Ca(BH_4_)_2_/THF system, which contributes to a decrease in the solvation energy of Ca^2+^ in the dual‐cation system. In addition, the liquid electrolyte Ca(BH_4_)_2_+LiBH_4_/THF was evaluated under the same test conditions (Figure S10, Supporting Information). A rapid decrease in overvoltage was observed at 0.1 mA cm^−2^, indicating a micro short circuit. This suggests that solidification suppresses dendritic growth. To compare the coulombic efficiency of calcium plating/stripping in GPE and liquid electrolytes, galvanostatic tests were performed using the same cell configuration as those used in CV. It is thought that there is almost no difference in coulombic efficiency between liquid and gel electrolytes (Figure S11a, Supporting Information). Details of plating/stripping behavior are discussed in the following section.

**Figure 5:**
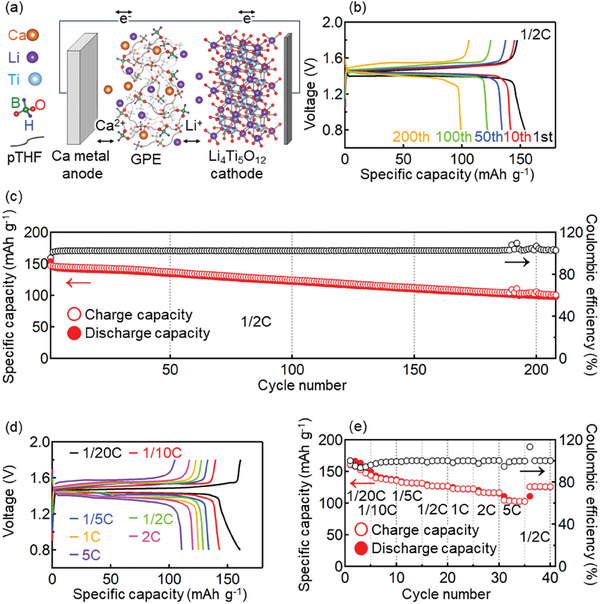
Charge/discharge test of dual‐cation GPE. a) Concept of dual‐cation battery (Ca|GPE|Li_4_Ti_5_O_12_). b) Charge/discharge curves recorded for different numbers of cycles at 0.5 C. c) Effects of cycling on specific capacity and Coulombic efficiency. d) Charge/discharge curves recorded at different C‐rates. e) Evolution of specific capacity and Coulombic efficiency during the rate capability test.

The concept of the battery with the dual‐cation GPE is shown in **Figure** **5**a. During discharge, Ca^2+^ ions dissolve from the Ca metal into the electrolyte, and Li^+^ ions in the GPE are intercalated into Li_4_Ti_5_O_12_. Conversely, Li^+^ ions are de‐intercalated from Li_4_Ti_5_O_12_ during charge, and Ca^2+^ ions are plated onto Ca metal. Li_4_Ti_5_O_12_ is an oxide widely used as an anode active material in lithium‐ion batteries. It has a spinel‐type structure that allows for the reversible intercalation/de‐intercalation of lithium ions.^[^ ^37^ ^]^

Figure 5b,c presents the results of the charge/discharge test performed for the Ca/Li_4_Ti_5_O_12_ cell with the dual‐cation GPE at 0.5 C, namely the corresponding charge/discharge curves and the effects of cycling on specific capacity and Coulombic efficiency. The first discharge capacity (154 mAh g^−1^) was 88% of the theoretical capacity of Li_4_Ti_5_O_12_ (175 mAh g^−1^). The discharge/charge cycling was performed for >200 cycles, with a discharge capacity of 99 mAh g^−1^ observed at the 200th cycle. The Coulombic efficiency after the second cycle exceeded 100%, which was ascribed to the oxidative decomposition of the BH_4_^−^ anions at the Li_4_Ti_5_O_12_ cathode during charging. While the Li_4_Ti_5_O_12_ cathode is known for its high cyclability, its discharge capacity degradation was faster in the dual‐cation GPE system. To discuss fast degradation, charge/discharge tests were conducted using three types of cells: Ca|Ca(BH_4_)_2_+LiBH_4_/THF|Li_4_Ti_5_O_12_, Ca|GPE with LiBH_4_|Li_4_Ti_5_O_12_, and Li|LiBH_4_/THF|Li_4_Ti_5_O_12_. The discharge capacities of Li|LiBH_4_/THF|Li_4_Ti_5_O_12_ (black circles) and Ca|dual‐cation GPE|Li_4_Ti_5_O_12_ (red circles) were higher and lower than those of Ca|Ca(BH_4_)_2_+LiBH_4_/THF|Li_4_Ti_5_O_12_ (blue circles), respectively (Figure S12, Supporting Information). This indicates that Ca^2+^ in the liquid electrolyte and gel polymer prevented Li^+^ from intercalating into Li_4_Ti_5_O_12_.

In the rate capability test, the charge/discharge capacity decreased with the increasing C rate (Figure 5d,e). However, there was a partial recovery in capacity when the rate returned to 0.5 C. The results show that stable, long‐life charging/discharging was achieved for the first time using a gel polymer electrolyte and Ca metal anode.

The average voltage during discharge/charge (1.47 V) did not match the value previously observed for a Li/Li_4_Ti_5_O_12_ cell (1.55 V),^[^ ^36b,c^ ^]^ which suggested that the anode reaction involved Ca metal and not Li metal (Figure S13, Supporting Information). Conversely, the voltage calculated from the difference between the standard electrode potentials of Li and Ca (≈0.17 V) equaled 1.38 V. This behavior was explained by the difference between the chemical potentials of Li^+^ and Ca^2+^ due to the different concentrations of the corresponding salts (LiBH_4_: 1.5M, Ca(BH_4_)_2_: 0.5M  in the original solution). In general, the chemical potential influences the electrode reaction potential. Therefore, in the dual‐ion battery system, the voltage cannot be calculated by simple estimation for each cation system. Similar voltage differences are often observed in dual‐cation battery systems (Mg^2+^/Li^+^ or Na^+^).^[^ ^38^ ^]^ The discharge capacity at the 10th cycle of GPE with LiBH_4_ is 141.6 mAh g^−1^, while that of GPE containing only Ca(BH_4_)_2_ is much lower at 4.9 mAh g^−1^ (Figure S14, Supporting Information), indicating that capacity by Ca^2+^ ions is negligible compared to Li^+^ ions. Therefore, discharge/charge is dominated by intercalation/de‐intercalation of Li^+^ ions, which dominate LTO. To confirm whether Ca plating/stripping occurs on the anode side, a galvanostatic measurement was performed for the Ca|dual‐cation GPE|Au cell at 0.1 mA cm^−2^. The black and red lines represent the voltage response of plating on and stripping from the gold electrode, respectively (Figure S11b, Supporting Information). The overvoltage of plating/stripping was approximately 50 mV versus Ca^2+^/Ca, which indicates that only Ca is plated onto and stripped from the gold electrode because of the difference between the standard electrode potentials of Li and Ca (≈0.17 V). The C rate in the battery test is 0.5C, which is converted to a current density of 0.073 mAcm^−2^. Therefore, it is thought that only Ca plating/stripping occurs on the anode side, even during the cycle test. The charge/discharge test results show that overvoltage increases with an increase in current density. However, the average voltage at the large current density of 0.7 mA cm^−2^ is lower than that of the Li|Li_4_Ti_5_O_12_ cell (Figure S15, Supporting Information). This indicates that lithium does not plate/strip on the anode side when charging/discharging at a large current density.

To compare the charging/discharging properties depending on the amount of THF content in the electrolyte, the charging/discharging tests at the same current density (0.073 mAcm^−2^) as shown in Figure 5b were performed using THF‐rich/poor dual‐cation GPE. THF‐rich/poor dual‐cation GPE was synthesized by substituting 1.5M LiBH_4_+0.5M Ca(BH_4_)_2_/THF for 1.5M Ca(BH_4_)_2_/THF solution in the synthesis method of THF‐rich/poor GPE. The results of full‐cell battery tests show that the overvoltage of THF‐rich/poor dual‐cation GPE is higher than that of dual‐cation GPE (Figure S16, Supporting Information). This is due to the larger overvoltage discussed in the galvanostatic tests.

Although our study paves the way for designing post‐lithium‐ion battery systems with GPEs, the narrow potential window of Ca(BH_4_)_2_ limits the range of compatible cathode materials. The realization of higher‐voltage solid‐state rechargeable Ca‐metal batteries requires the development of electrolytes with excellent oxidation stabilities, e.g., those based on Ca(CB_11_H_12_)_2_.^[^ ^7^, ^29^ ^]^ In the previous report of the Mg electrolyte system using the cross‐linking reaction of borohydride anions and pTHF, the gel electrolyte exhibits high electrochemical properties by adding a different type of anion with excellent electrochemical properties.^[^ ^30^ ^]^ In the future, we will synthesize and evaluate a novel GPE with high oxidation stability by combining Ca(CB_11_H_12_)_2_ as the electrolyte salt and Ca(BH_4_)_2_ as the cross‐linking agent. Although the ionic conductivity was low in this system, it is expected that the ionic conductivity can be significantly improved by increasing the ion concentration by adding a salt containing weakly coordinating (CB_11_H_12_) anions.

## 3 Conclusion

A Ca^2+^‐conductive GPE compatible with Ca‐metal anodes was synthesized through a cross‐linking reaction between BH_4_^−^ from Ca(BH_4_)_2_ and terminal ─OH groups of pTHF. This electrolyte showed high stability against Ca‐metal anodes, a low overpotential in a galvanostatic test, and an ionic conductivity of *σ* = 1.92 × 10^−5^ S cm^−1^ according to EIS, thus showing significant potential for Ca‐metal batteries. LSV tests suggested that BH_4_^−^ anions decomposed at voltages above 2.2 V versus Ca^2+^/Ca. Charge/discharge tests performed for >200 cycles at 0.5 C using a dual‐cation (Ca^2+^ and Li^+^ from corresponding borohydrides) cell with a Ca‐metal anode and Li_4_Ti_5_O_12_ cathode indicated that the addition of LiBH_4_ improved Ca plating/stripping behavior. This study indicates the feasibility of practical Ca‐metal batteries with polymer electrolytes and paves the way for future use of electrolyte salts with high oxidation stability.

## 4 Experimental Section

Air‐sensitive materials were prepared and handled in an atmosphere of dry argon using a glovebox and Schlenk techniques.

### Synthesis of Ca(BH_4_)_2_ based‐gel polymer electrolyte

Solutions of Ca(BH_4_)_2_ (1.5 m) and pTHF (1.0 g mL^−1^) in THF, obtained by dissolving Ca(BH_4_)_2_·2THF (321 mg, Sigma‐Aldrich) and pTHF (1.0 g, Sigma‐Aldrich) in THF (1 mL, Sigma‐Aldrich), respectively, were supplemented with molecular sieves (diameter: 2.9–3.5 mm, FUJIFILM) and left to stand overnight. Subsequently, the two solutions were combined, and the mixture was stirred for 10 min. After stirring, the solution solidified enough to afford a GPE. The reaction is allowed to proceed until no H_2_ gas is emitted from the GPEs.

1.5M Ca(BH_4_)_2_/THF solution, pTHF/THF, and THF solvent were measured at a 1: 1: 2 volume ratio, mixed, and stirred to prepare a mixed solution. This mixed solution solidified and became a gel‐polymer electrolyte, represented as THF‐rich GPE. A solution of 2 g of pTHF dissolved in 1 ml of THF was prepared and represented as a THF‐poor solution. This solution, 1.5M Ca(BH_4_)_2_/THF solution, and THF‐poor solution were measured at a volume ratio of 1:1, mixed, and stirred to prepare a mixed solution. This mixed solution solidified and became a gel‐polymer electrolyte, represented as THF‐poor GPE. Figure S17 (Supporting Information) shows the volume ratio of each solution for the synthesis of gel‐polymer electrolytes.

For dual‐cation GPE synthesis, Ca(BH_4_)_2_·2THF (107 mg) and LiBH_4_ (33 mg, Sigma‐Aldrich) were dissolved in THF (1 mL) to afford a 0.5M  Ca(BH_4_)_2_ + 1.5M  LiBH_4_/THF solution, which was processed as described earlier.

### Characterization of GPE

The terminal ─OH groups of pTHF were detected by FT‐IR (Nicolet iN 10, Thermo Scientific) using a diamond cell. This cell absorbed infrared light with wavenumbers of 1667–2500 cm^−1^, so this range was excluded from the acquired spectra. The GPE structure was probed by Raman spectroscopy (DXR, Thermo Scientific) and XRD (X'PERT Pro, PANalytical; Cu K_α_ radiation (*λ* = 1.5406 Å for K_α1_ and 1.5444 Å for K_α2_). ^43^Ca, ^11^B, and ^1^H NMR measurements were carried out in 5‐mm glass tubes at ambient temperature and frequencies of 33.659, 160.45, and 500.13 MHz, respectively, using a Bruker Avance 500‐MHz spectrometer. ^43^Ca, ^1^H, and ^11^B shifts were referenced to 1 m Ca(NO_3_)_2_ in D_2_O (*δ*_iso_ = 0.0 ppm), 2 vol% tetramethylsilane in CDCl_3_ (*δ*_iso_ = 0.0 ppm), and H_3_BO_3_ (*δ*_iso_ = 19.5 ppm) as external standards, respectively.^43^Ca NMR spectra were acquired using a spin echo pulse sequence with 2k acquisitions. The Dmfit program developed by Dominique Massiot^[^ ^39^ ^]^ was used to fit ^1^H NMR spectra.

### Electrochemical Analysis of GPE

Electrochemical cells were assembled in a dry argon‐filled glovebox by soaking glass fiber in non‐solidified electrolyte precursor solutions and sandwiching it between electrodes. The electrochemical evaluation was carried out after the completion of the solidification reaction. Ca metal for electrochemical tests was polished to a metallic luster. All electrochemical analyses and battery tests were conducted using a two‐electrode cell at room temperature. The stability of GPEs against Ca metal was evaluated by galvanostatic measurements using Ca|GPE|Ca symmetric cells. A constant current density (±0.05 mA cm^−2^) was applied to the cells every 30 min, and the voltage change was recorded. The ionic conductivity was probed by electrochemical impedance spectroscopy (EIS). A sine‐wave voltage with an amplitude of 150 mV was applied to a Mo symmetric cell 1 h after assembly in the frequency range of 1 MHz to 4 Hz. Ionic conductivity *σ* was calculated as *σ* = *l*/*SR*, where *l* is the thickness of the glass fiber, *S* is the area of the electrode, and *R* is the resistance extracted from the EIS plot. The reversibility of Ca plating and stripping was examined by CV measurements, which were conducted using an electrochemical cell (Ca|GPE|Au) at a scan rate of 10 mV s^−1^ within a voltage range of −1 to 2 V versus Ca^2+^/Ca. The electrolyte oxidation stability was evaluated by LSV at a scan rate of 1.0 mV s^−1^ within a voltage range of 0–4 V versus Ca^2+^/Ca using a cell configuration identical to that used for CV. Molybdenum and aluminum electrodes were used to investigate differences in oxidation stability due to electrodes other than gold. To evaluate stability against Ca metal at different current densities, a constant current density (10, 20, 50, 100, 200, and 500 µA cm^−2^) was applied to a Ca|GPE or LiBH_4_‐supplemented GPE|Ca symmetric cell for 30 min every 10 times.

### Battery Tests Using Dual‐Cation Electrolyte System

Battery tests were conducted using the dual‐cation GPE, Ca metal as the anode, and Li_4_Ti_5_O_12_ (Sigma‐Aldrich) composite as the cathode. The purity of the purchased Li_4_Ti_5_O_12_ was confirmed with XRD analysis (Figure S18, Supporting Information). The Li_4_Ti_5_O_12_ composite was prepared by mixing Li_4_Ti_5_O_12_ powder, acetylene black (Denka Black Li, Li‐435, Denka), and poly(vinylidene difluoride) (KF Polymer L#1120, Kureha) in a 90:6:4 mass ratio in *N*‐methyl‐2‐pyrrolidone, applying this slurry over an aluminum current collector, and heating at 353 K under vacuum for 12 h. The average mass of the prepared cathodes was 2.46 mg (*n* = 10). Charge/discharge tests were carried out at 0.5 C and cutoff voltages of 1.8–0.8 V. Rate capability tests were conducted at 0.05, 0.10, 0.20, 0.50, 1.0, 2.0, and 5.0 C every five cycles.

## Conflict of Interest

The authors declare no conflict of interest.

## Supporting information

Supporting Information

## Data Availability

The data that support the findings of this study are available from the corresponding author upon reasonable request.
