# Supplementary material for: Complex Hydride‐Based Gel Polymer Electrolytes for Rechargeable Ca‐Metal Batteries
Source: Adv Sci (Weinh). 2024 Jul 3;11(33):2308318. doi: 10.1002/advs.202308318 (PMC11434029; doi:10.1002/advs.202308318)
Supplement: Supplementary file 1 — Supporting Information [file ADVS-11-2308318-s001.pdf]

ADVANCED SCIENCE
Open Access

Supporting Information

for *Adv. Sci.*, DOI 10.1002/advs.202308318

Complex Hydride-Based Gel Polymer Electrolytes
for Rechargeable Ca-Metal Batteries

Takara Shinohara, Kazuaki Kisu, Arunkumar Dorai, Kenji Zushida, Hiroshi Yabu, Shigeyuki Takagi and Shin-ichi Orimo**

Supporting Information

Complex hydride-based gel polymer electrolytes for rechargeable Ca metal batteries

Takara Shinohara, Kazuaki Kisu, Arunkumar Dorai, Kenji Zushida, Hiroshi Yabu, Shigeyuki Takagi, and Shin-ichi Orimo**

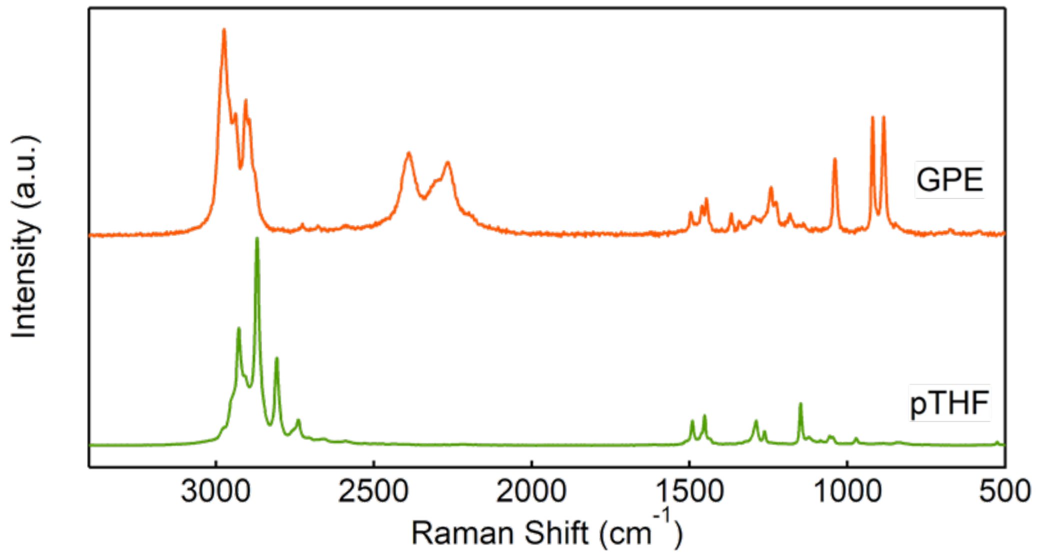

Figure S1. Raman spectra of synthesized gel polymer electrolyte (GPE; orange) and poly(tetrahydrofuran) (pTHF; green).

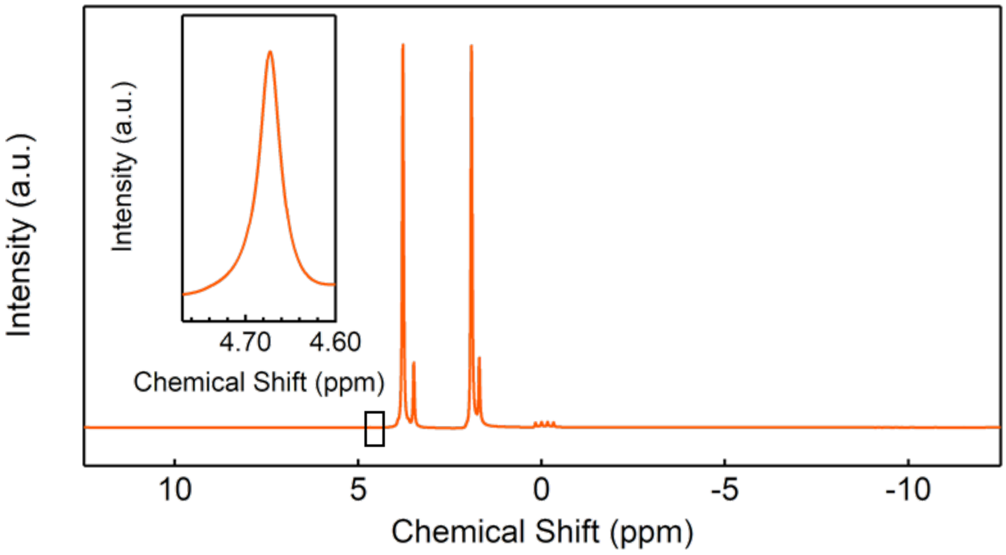

Figure S2. ^{1}H NMR spectrum of GPE after polymerization.

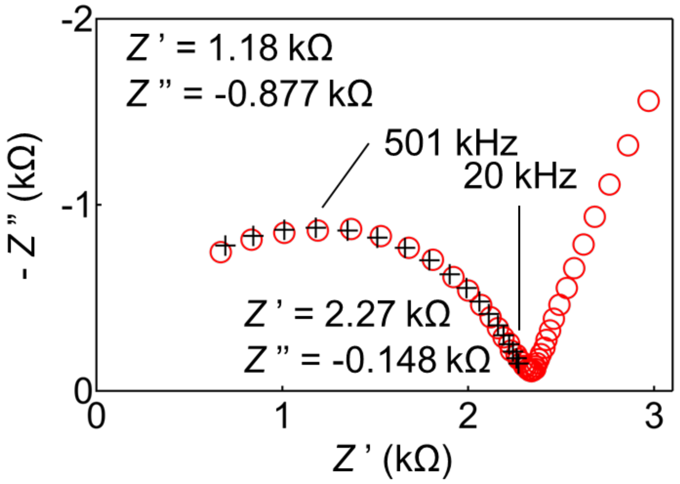

Figure S3. Enlarged view of inset in Figure 3a

Table S1. Fitting parameters of the equivalent circuit

Element	Value
Resistance	2.33×10^{3}
CPE-T	1.90×10^{-9}
CPE-P	0.823

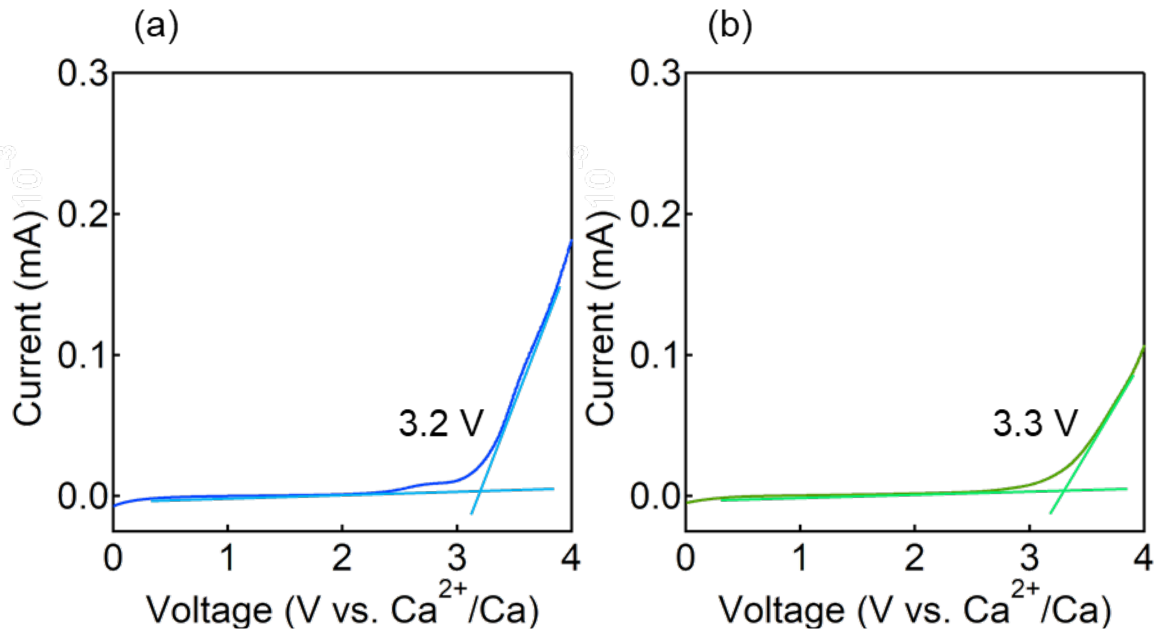

Figure S4. Linear sweep voltammograms of the (a) Ca|Mo and (b) Ca|Al cells at a scan rate of 1 mV s^{-1}.

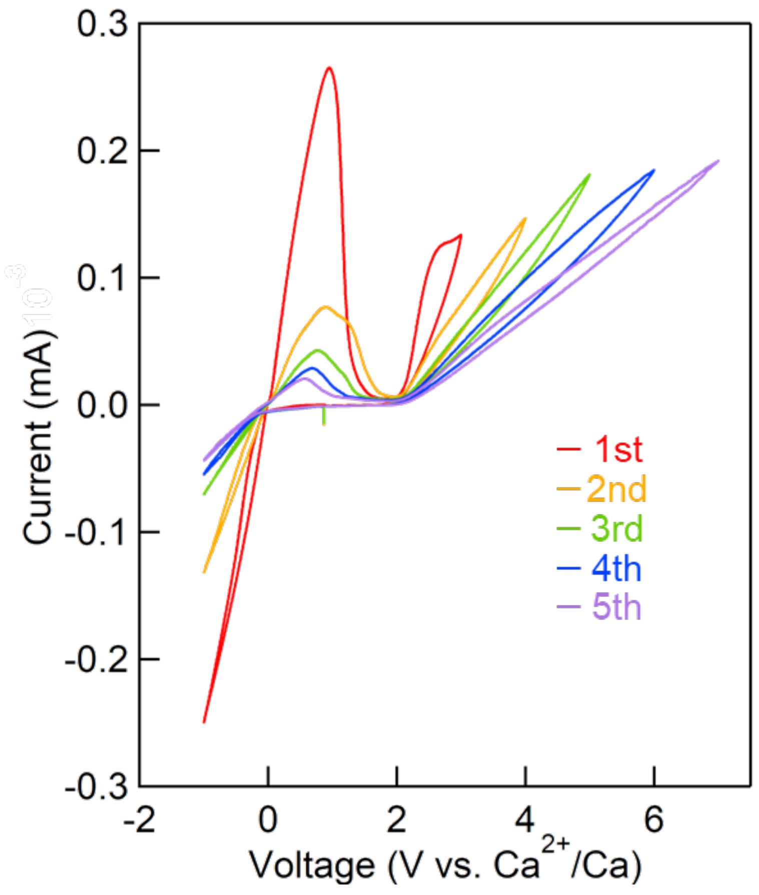

Figure S5. Cyclic voltammograms of GPE (1st: −1 to 3 V, 2nd: −1 to 4 V, 3rd: −1 to 5 V, 4th: −1 to 6 V, and 5th: −1 to 7 V) at a scan rate of 1 mV s^{-1}.

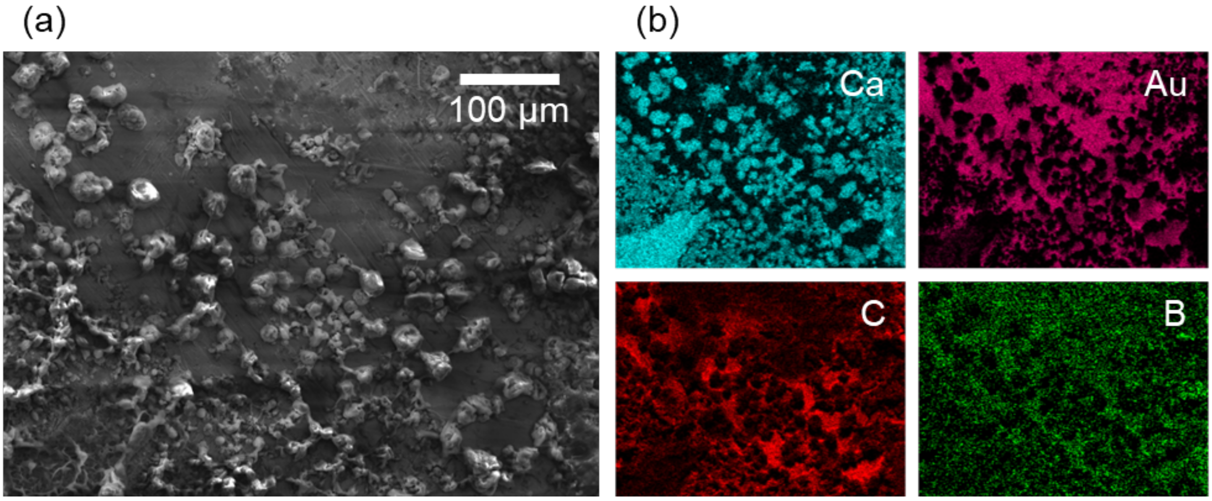

Figure S6. (a) Scanning electron microscopy image and (b) corresponding elemental mappings (Ca, Au, C, B) of the deposit formed on the gold electrode.

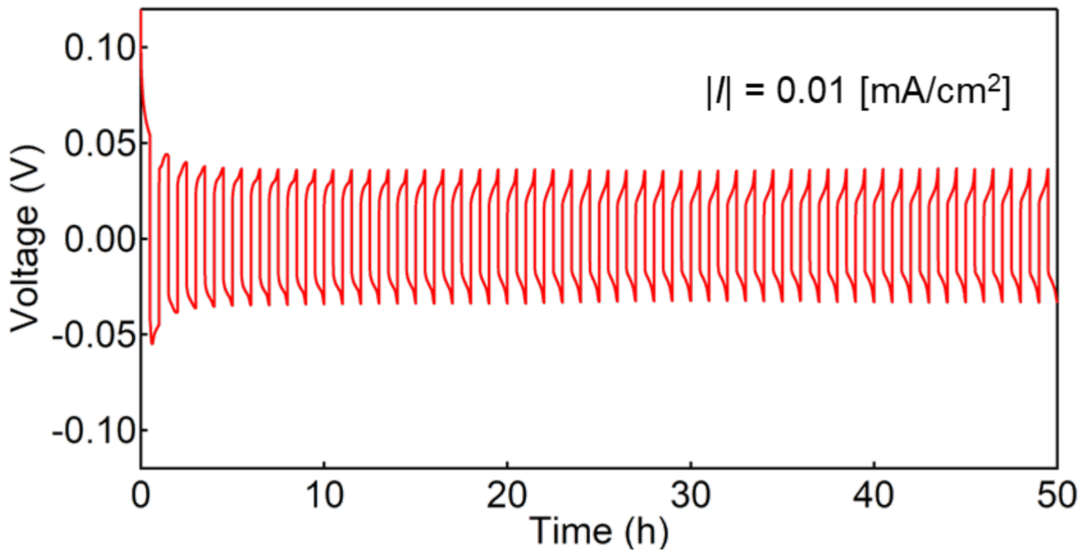

Figure S7. Galvanostatic curves (|*I*| = 0.01 mA cm^{-2}, Ca|GPE|Ca).

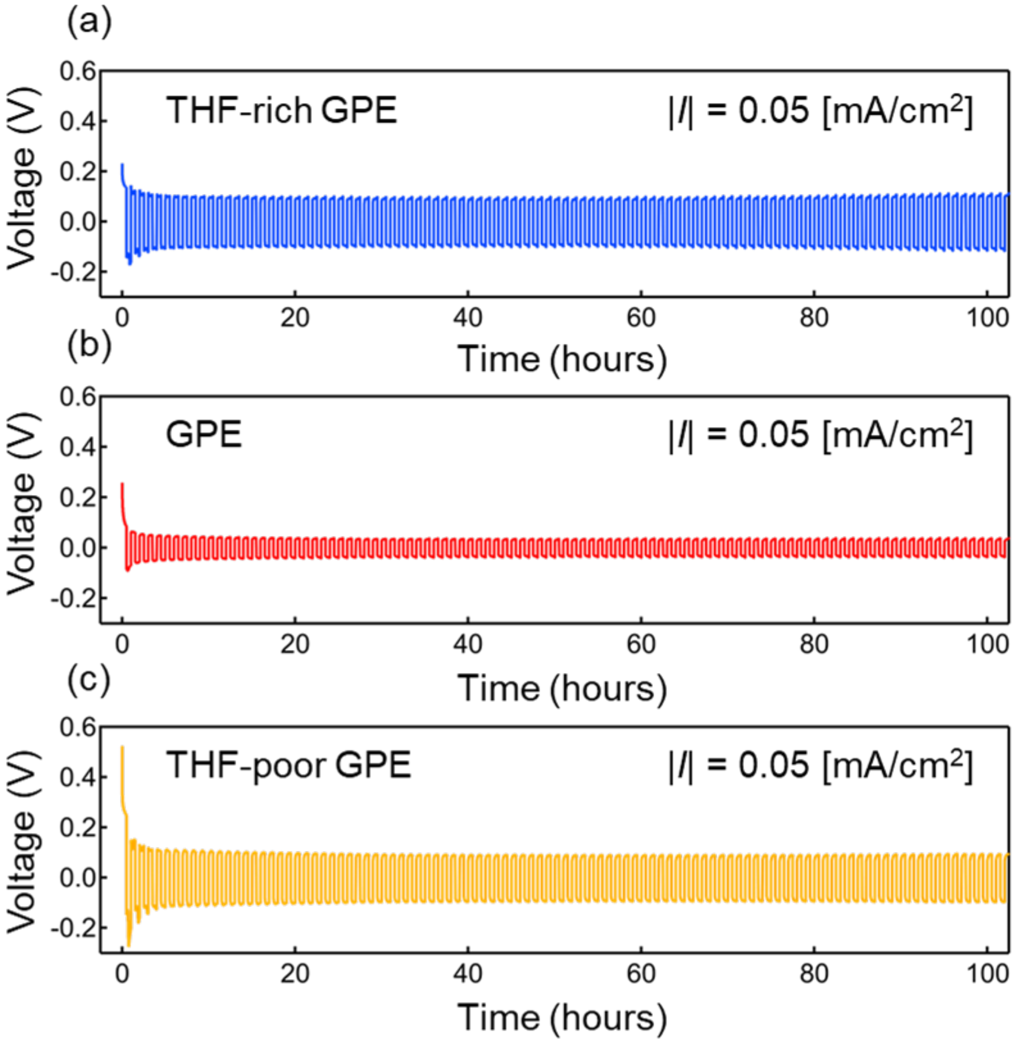

Figure S8. Difference in voltage response depending on THF content (a)THF-rich GPE (b) GPE (c) THF-poor GPE

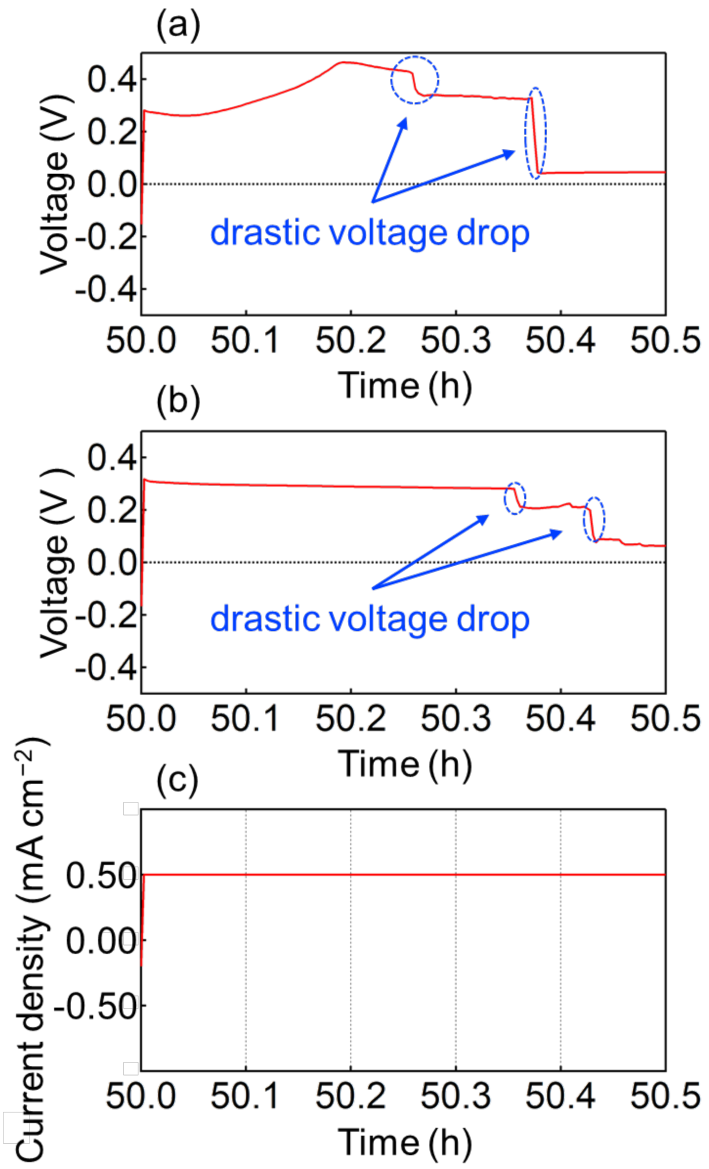

Figure S9. (a) Voltage changes of GPEs at various current densities (enlarged map of the red line of Figure 4b at 50–50.5 h). (b) Voltage changes in another electrochemical cell under the same conditions. (c) Current density.

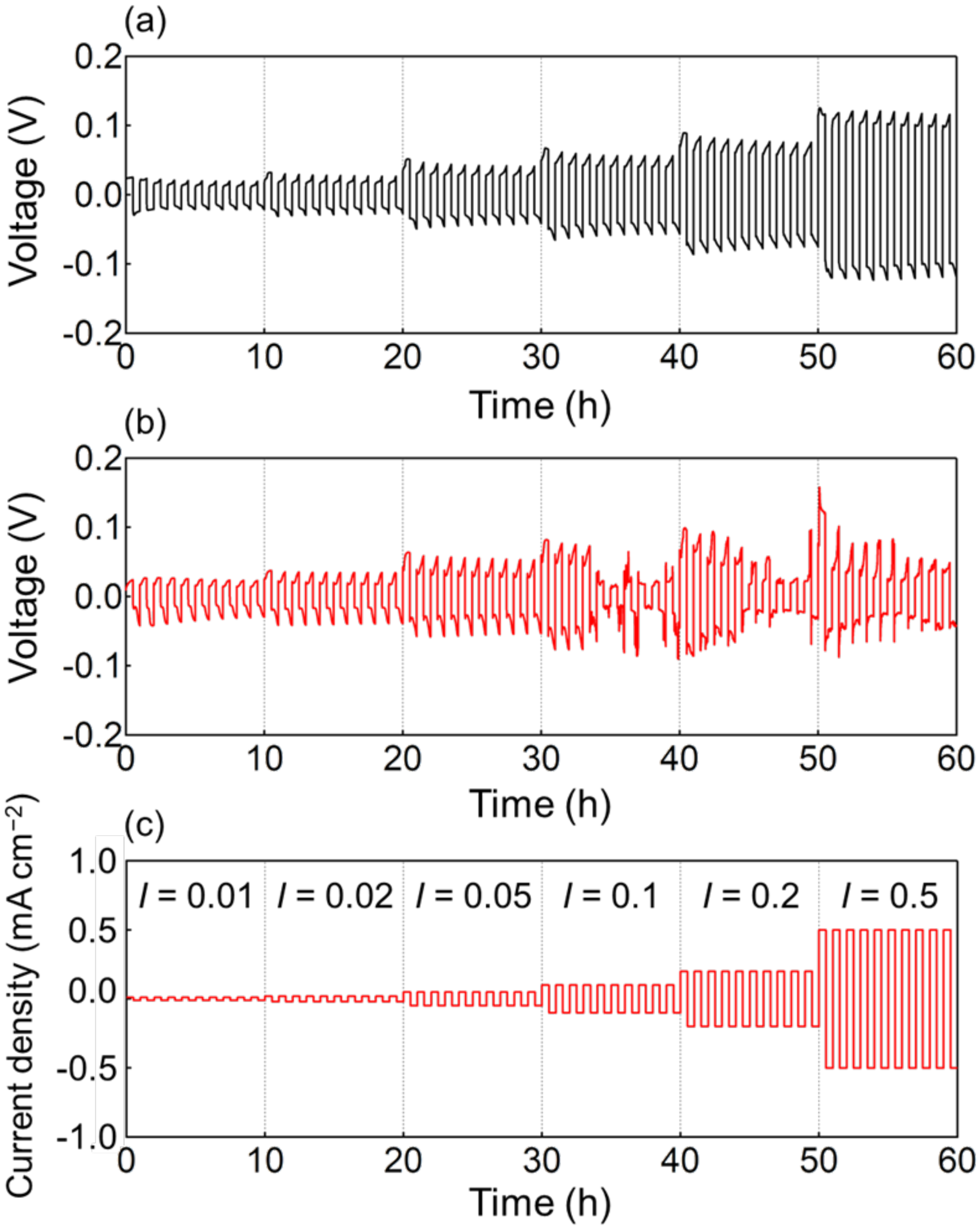

Figure S10. Voltage changes at various current densities of (a) dual-cation GPEs and (b) $Ca(BH_4)_2$+$LiBH_4$/THF. (c) Applied current density.

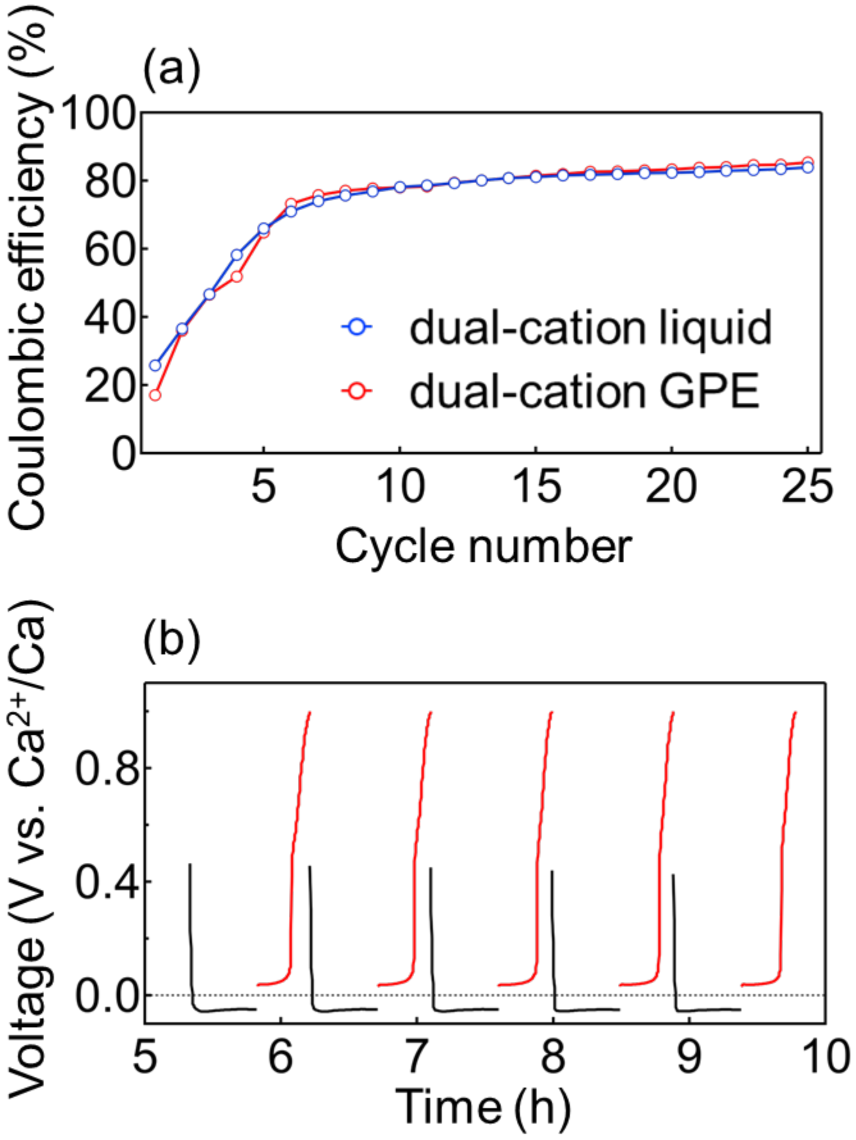

Figure S11. (a) Coulombic efficiency of dual-cation liquid electrolyte and dual-cation GPE (b) Voltage response of plating/stripping in Ca|dual-cation GPE|Au cell (black line: plating, red line: stripping).

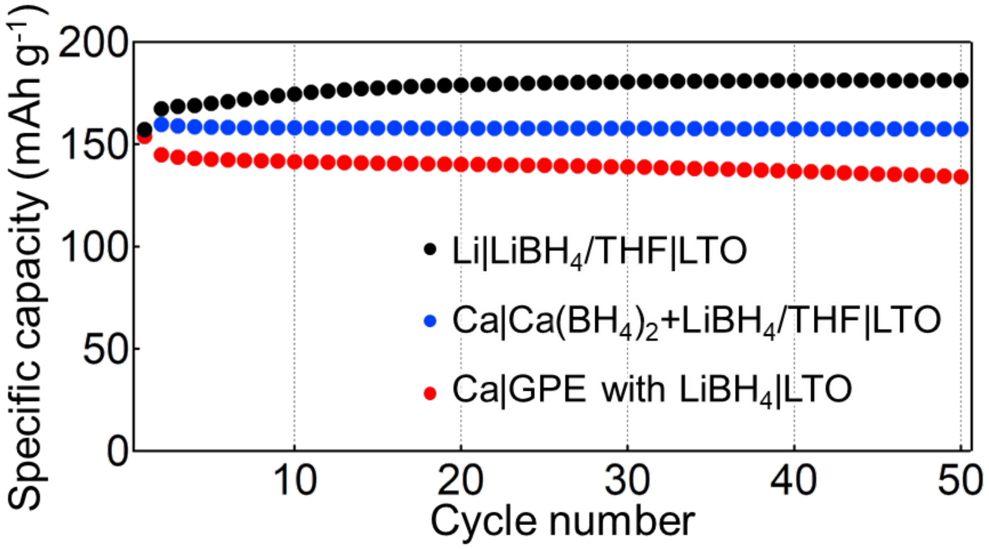

Figure S12. Discharge capacity of three types of electrolytes.

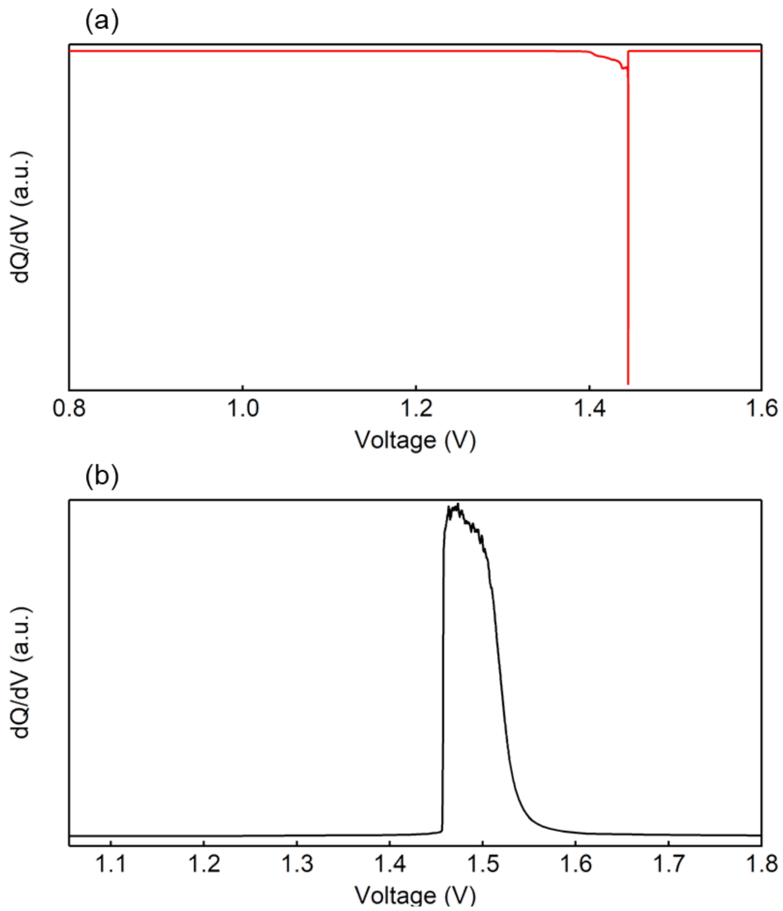

Figure S13. dQ/dV curves at the 10th cycle of (a) discharging and (b) charging.

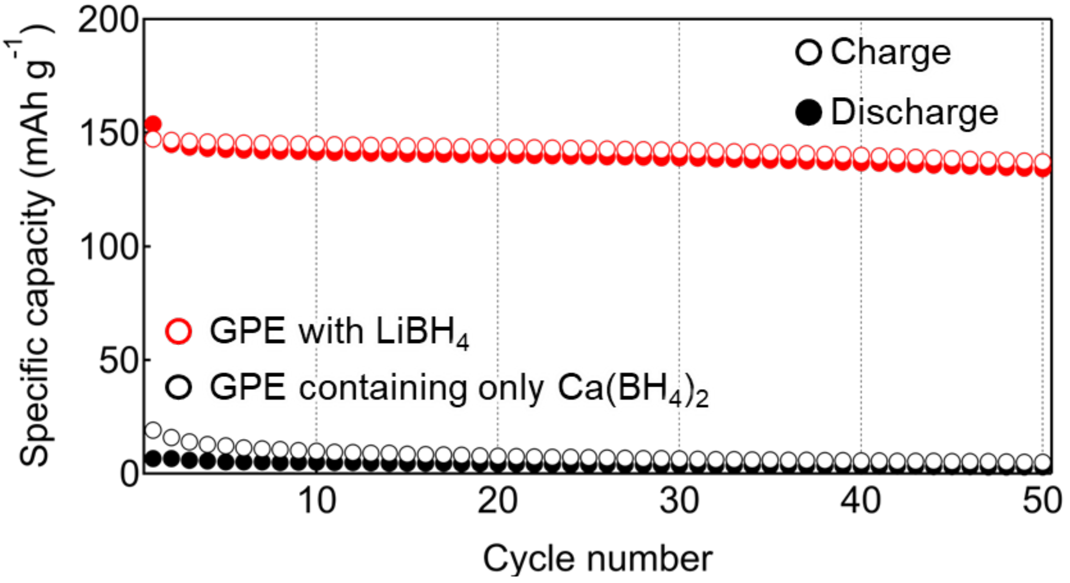

Figure S14. Specific capacity of Ca|GPE with $Li_4Ti_5O_{12}$ and containing only $Ca(BH_4)_2$.

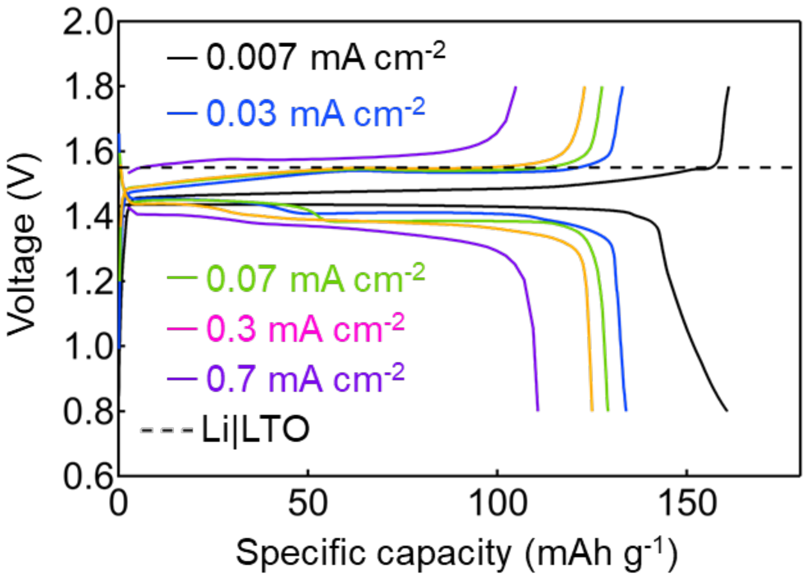

Figure S15. Charge/discharge curves recorded at various current densities.

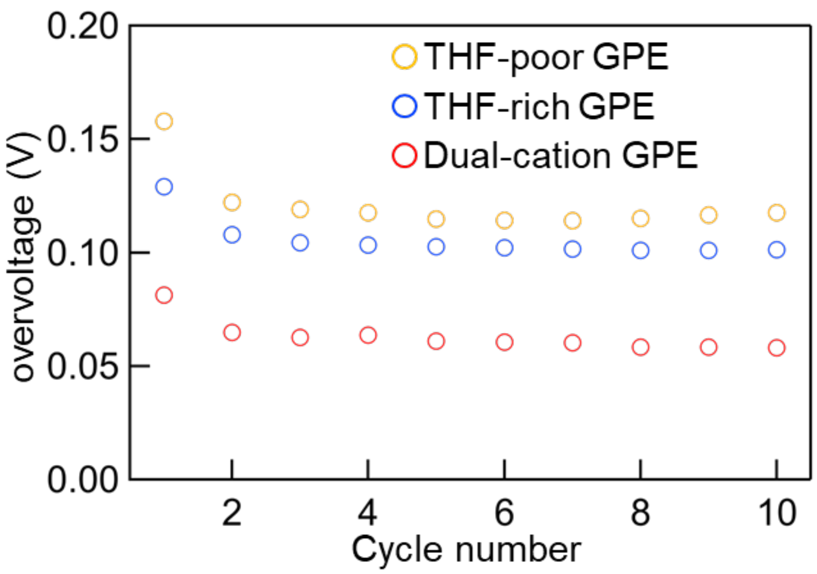

Figure S16. Overvoltage of full cell using THF-rich/poor GPEs in charge/discharge test at 73 μA cm^{-2}.

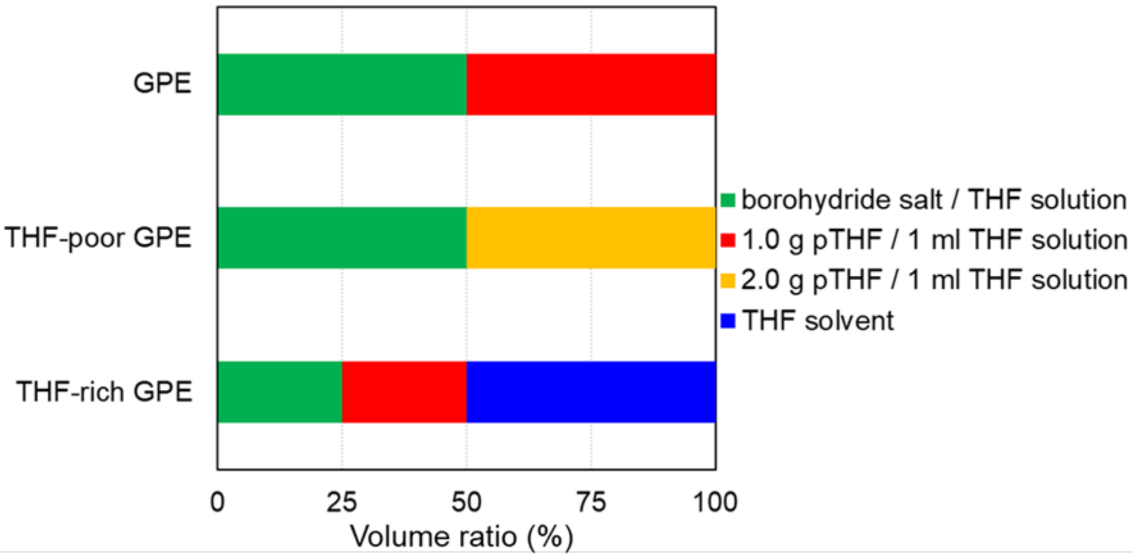

Figure S17. Volume ratio of each solution for synthesis of GPEs.

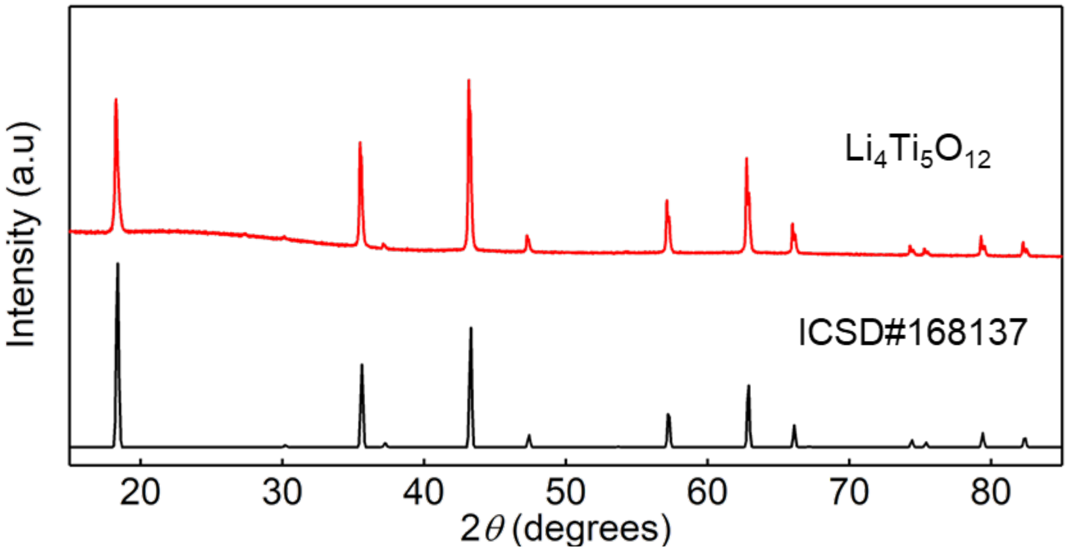

Figure S18. XRD patterns ($Li_4Ti_5O_{12}$ for cathode material and reference).
